# Role of nasal high-frequency oscillatory ventilation in a premature infant with severe bronchopulmonary dysplasia

**DOI:** 10.1016/j.rmcr.2025.102226

**Published:** 2025-05-02

**Authors:** Akiho Ueda-Kuramochi, Kazumi Morisawa, Takeshi Arimitsu, Kazuma Shimura, Kaori Hara-Isono, Takane Kin, Mariko Hida

**Affiliations:** Department of Pediatrics, Keio University School of Medicine, 35 Shinanomachi, Shinjuku-ku, Tokyo, 160-8582, Japan

**Keywords:** Bronchopulmonary dysplasia, Extremely low birth weight, Extremely preterm, Extubation, Nasal high-frequency oscillatory ventilation

## Abstract

To prevent the worsening of bronchopulmonary disease (BPD), early extubation is desirable. However, in extremely preterm infants, BPD tends to become severe, making early extubation difficult and leading to prolonged intubation. Even if the intubation period is prolonged, feasible respiratory strategies for extubation in extremely preterm infants during the chronic phase of severe BPD are necessary. In preterm infants, nasal high-frequency oscillatory ventilation (NHFOV) can support breathing after extubation immediately after birth, but whether NHFOV is effective as respiratory support after extubation in the chronic phase of severe BPD in extremely preterm infants is unclear. Especially for extremely preterm births or infants with extremely low birth weights, early extubation is difficult. Although such infants' postmenstrual age and weight increase during long-term ventilator support, their respiratory function is very poor compared with that of preterm infants born at a gestational age equivalent to such infants' postmenstrual age owing to substantial lung damage caused by the ventilator. For this reason, extubation in the chronic phase of BPD may also be challenging. In this report, we describe a case of a marginally viable infant who was born at 23 weeks’ gestation weighing 374 g, required 2 months of intubation after birth owing to severe BPD, and was successfully extubated using NHFOV. This case report suggests that NHFOV may be an effective respiratory strategy for very low birth weight infants with severe BPD.

## 1 Introduction

Severe bronchopulmonary dysplasia (BPD) affects 8 % of extremely preterm infants [1]. It is associated with high rates of mortality and neurodevelopmental disorders [2]. Early extubation is important to prevent the progression of BPD [3]. Extremely preterm infants are at a high risk of BPD progression and need to be extubated during the early phase after birth. However, because their respiratory function often declines during that phase, they may require long-term intubation. Therefore, new respiratory strategies that ensure the earliest possible extubation, even if intubation is prolonged, are necessary. Nasal high-frequency oscillatory ventilation (NHFOV) provides effective post-extubation support soon after birth [[4], [5], [6], [7], [8], [9]]. However, little research has been performed on the effectiveness of NHFOV as a post-extubation strategy during the chronic phase of severe BPD in extremely preterm infants. Here, we report the case of an extremely preterm infant with severe BPD and an extremely low birth weight who underwent successful extubation using NHFOV after 2 months of intubation. This report is unique because the patient had the lowest gestational age (23 weeks), lowest birth weight (374 g), and also the longest intubation period (2 months), indicating that he had very serious BPD. Although studies on the use of NHFOV as respiratory support after extubation in extremely preterm infants within 2 weeks of birth have been reported, almost none on the effectiveness of NHFOV as a respiratory-support strategy after extubation in extremely low birth weight infants who have developed severe BPD and have been intubated for a long period have been reported. This case suggests that NHFOV may be an effective respiratory-support strategy for extremely preterm infants after extubation in the chronic phase of severe BPD.

## 2 Case report

The patient, a boy, was born via emergency cesarean section because of placental abruption at 23 weeks 2 days of gestation. He exhibited no clinical signs of chorioamnionitis. Weak spontaneous breathing necessitated intubation and endotracheal surfactant administration. At birth, the following measurements were obtained: 1-min and 5-min Apgar scores of 3 and 5, respectively; weight, 374 g (standard deviation [SD], −2.59; percentile, 0.5); height, 29 cm (SD, −0.03; percentile, 48.7); and head circumference, 20.2 cm (SD, −0.43; percentile, 33.3). Parenteral nutrition commenced on day 0. Ileal atresia caused an ileal perforation and generalized peritonitis on day 9, requiring an ileostomy.

**Fig. 1:**
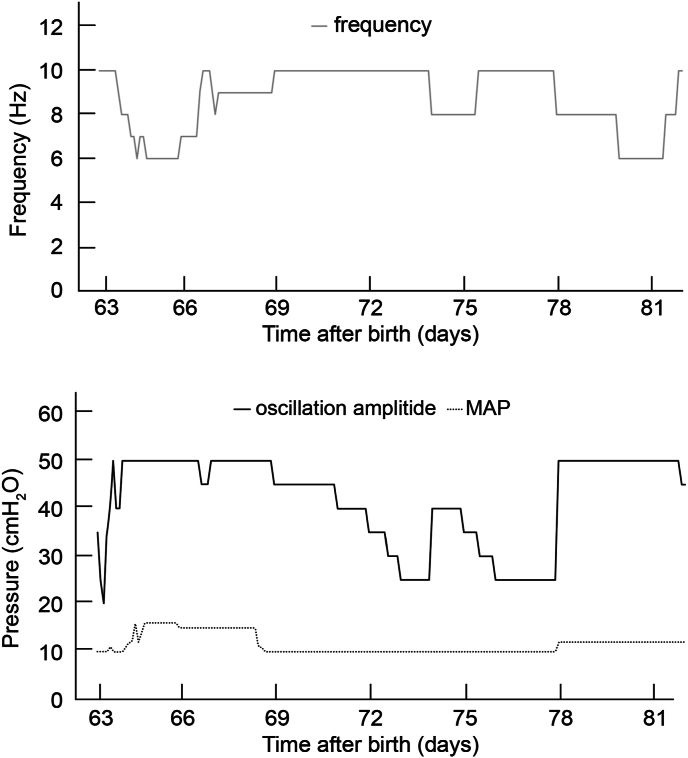
Nasal high-frequency oscillatory ventilation (NHFOV) settings used after extubation. The solid black, dotted, and solid gray lines indicate oscillation amplitude, mean airway pressure (MAP), and frequency output, respectively.

Because the patient had an extremely low gestational age and birth weight, indicating that he was very premature, he experienced respiratory distress from birth, which progressed immediately after birth. Therefore, he could not be extubated early and required long-term ventilator management. Before extubation, he was managed with ventilation synchronized-flow trigger intermittent mandatory ventilation with a fraction of inspired oxygen (FiO_2_) of 0.21–0.30, peak inspiratory pressure of 16–18 cmH_2_O, pressure support of 10–15 cmH_2_O, positive end-expiratory pressure of 5 cmH_2_O, and respiratory rate of 45–50 breaths/min. He was extubated on day 63 and received NHFOV as respiratory support. The two-hole prongs used for NHFOV did not fit our patient; hence, a mask was used instead. Oscillation frequency and amplitude were adjusted for a CO_2_ level of 35–45 mmHg, and the inspiratory time was set at 50 % (1:1). The mean airway pressure (MAP) and FiO_2_ were regulated to maintain a peripheral oxygen saturation of 85 %–95 %. Sigh breaths were delivered at a peak pressure of 6–8 cmH_2_O above the MAP (frequency: 3 breaths/min, inspiratory time: 0.65 s; Fig. 1). NHFOV was changed to nasal intermittent positive pressure ventilation (NIPPV) on day 82, which in turn was switched to high-flow nasal therapy on day 96. Ileostomy closure on day 173 required temporary intubation, immediate extubation postoperatively, and reinitiation of high-flow nasal therapy. Oxygenation was managed with a nasal cannula on days 189–196.

Corticosteroid therapy for BPD comprised three courses of hydrocortisone and two of dexamethasone. Hydrocortisone was administered as follows: the first course (from day 1, for 10 days) at 1 mg/kg/day for 7 days, followed by 0.5 mg/kg/day for 3 days; the second course (from day 16, for 22 days) at 5 mg/kg/day for 7 days, followed by 3.75 mg/kg/day for 5 days, 2.5 mg/kg/day for 5 days, and 1.25 mg/kg/day for 5 days; and the third course (from day 63) at 1.5 mg/kg/day, with gradual tapering until complete withdrawal on day 124. Dexamethasone was administered on days 35–45 and 64–74, starting at 0.15 mg/kg/day (3 days), followed by 0.10 mg/kg/day (3 days), 0.05 mg/kg/day (2 days), and 0.02 mg/kg/day (2 days). Additional steroids were administered several times as treatment for circulatory failure and other conditions.

**Fig. 2:**
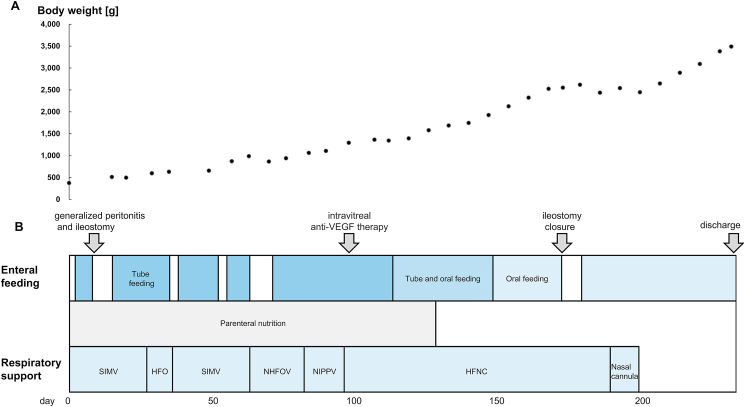
Summary of treatment progress during hospitalization. The upper graph shows weight progression. The lower graph shows the events, feeding, nutrition, ventilatory support types, and intubation tube size. HFNC, high-flow nasal cannula; HFOV, high-frequency oscillatory ventilation; NHFOV, nasal high-frequency oscillatory ventilation; NIPPV, nasal intermittent positive pressure ventilation; SIMV, synchronized intermittent mandatory ventilation; VEGF, vascular endothelial growth factor.

His respiratory status remained stable under general anesthesia throughout the ileostomy closure, and he was not given any sedatives or analgesics except during surgery. Magnetic resonance imaging of his head on day 207 revealed no abnormalities, and the automated auditory brainstem response test revealed normal responses bilaterally. The patient was discharged on day 233, weighing 3488 g (Fig. 2).

## 3 Discussion

Compared with the patients in related previous studies, our patient had the lowest gestational age, lowest birth weight, and longest intubation period [[4], [5], [6], [7]]. This case suggests that NHFOV is a feasible respiratory strategy for extubation in extremely preterm infants in the chronic phase of severe BPD.

Survival rates of extremely preterm and extremely low-birth-weight infants have recently improved [1]. Hence, the incidence of severe BPD has increased. Although early extubation is essential to prevent severe BPD as intubation can cause lung damage, BPD tends to become more severe in such infants during the early phase after birth, complicating early extubation. In turn, prolonged intubation exacerbates BPD, which requires ventilator management and further prolongs intubation. Thus, extubation must be expedited, even after prolonged intubation, to prevent progression of BPD, and new post-extubation strategies are necessary for extremely preterm infants in the chronic phase of severe BPD.

NHFOV is highly effective for removing CO_2_, resolving patient–ventilator dyssynchrony with spontaneous breathing, and improving oxygenation [8]. NHFOV is more effective than nasal continuous positive airway pressure ventilation (NCPAP)in reducing of CO_2_ partial pressure and reintubation rates after extubation during the early phase after birth [4]; in addition, NHFOV can reduce the ventilator duration compared with NIPPV [5]. Reintubation in extremely preterm infants is also associated with increased rates of BPD and mortality [9]; therefore, extubation and subsequent optimal support are crucial, especially for infants with severe BPD. NHFOV is considered suitable for post-extubation respiratory support of extremely preterm infants with severe BPD at high risk of reintubation [8]. However, whether NHFOV is effective as respiratory support after extubation of extremely preterm infants with severe BPD who require long-term intubation has been examined in only a few studies.

Patients in previous studies [[4], [5], [6], [7]] who underwent extubation during the early phase after birth had higher gestational ages and birth weights and much shorter intubated periods than our patient. However, the oscillation amplitude, MAP, and frequency output of NHFOV could be managed with settings comparable to those reported previously. Nasal masks are more flexible than nasal prongs, and the flexibility may dampen the vibration in NHFOV [10], but in this case, the patient could be managed using the same settings as in previous studies [[4], [5], [6], [7]] despite the use of a nasal mask. Although previous reports did not mention the sigh pressure setting, we used the setting that is normally used for high-frequency-oscillatory ventilation and confirmed that it is safe and valid even for NHFOV [11]. Abdominal distension and upper airway obstruction caused by highly viscous secretions (side effects of NHFOV) [4] were not observed in this case. However, during NHFOV, the alarm frequently sounded when the oscillation amplitude fluctuated because of a temporary leak, indicating the need for device improvement.

To date, early NHFOV has not been demonstrated to have long-term beneficial effects, and whether infants with advanced or established BPD can be weaned from ventilators in the chronic phase needs to be investigated in future, prospective, randomized clinical trials. A recent comparative study of NHFOV and NIPPV demonstrated potential benefits of NHFOV without an increased risk of complications [12], and no adverse effects occurred in our case. Therefore, in marginally viable infants for whom extubation is difficult owing to severe BPD, as in our case, NHFOV should be considered as treatment option. However, one limitation of our study is that NHFOV might not have been the only potentially successful post-intubation strategy in this case, as other strategies were not tested. Considering that severe BPD is phenotypically diverse, with a wide range of clinical manifestations, NCPAP may also be successful; however, this approach was not investigated in our case. In conclusion, the treatment strategy reported here may be useful for extubation of critically ill and marginally viable infants with severe BPD who require prolonged intubation. These findings may contribute to further development in neonatal medicine.

## CRediT authorship contribution statement

**Akiho Ueda-Kuramochi:** Writing – original draft, Investigation, Data curation, Conceptualization. **Kazumi Morisawa:** Writing – original draft, Investigation, Data curation, Conceptualization. **Takeshi Arimitsu:** Supervision, Project administration, Funding acquisition, Data curation, Conceptualization. **Kazuma Shimura:** Writing – review & editing. **Kaori Hara-Isono:** Writing – review & editing. **Takane Kin:** Writing – review & editing. **Mariko Hida:** Writing – review & editing.

## Study approval statement

The Clinical and Translational Research Center - Keio University did not require ethics approval because our study was a case report.

## Consent to publish statement

Written informed consent was obtained from the patient's parents for publication of this case report.

## Data availability statement

All data generated or analyzed during this study are included in this article. Further enquiries can be directed to the corresponding author.

## Funding sources

This work was supported by 10.13039/501100001700MEXT 10.13039/501100001691KAKENHI (grant no. JP 19K12734 to TA). The funder had no role in the design, data collection, data analysis and interpretation, writing of the report, or decision to submit the article for publication.

## Declaration of competing interest

The authors declare the following financial interests/personal relationships which may be considered as potential competing interests: Takeshi Arimitsu reports financial support was provided by 10.13039/501100001700MEXT 10.13039/501100001691KAKENHI (grant no. JP 19K12734). Takeshi Arimitsu reports a relationship with 10.13039/501100001700MEXT 10.13039/501100001691KAKENHI that includes: funding grants. If there are other authors, they declare that they have no known competing financial interests or personal relationships that could have appeared to influence the work reported in this paper.
